# Risk factors for repeated recurrence of cerebral aneurysms treated with endovascular embolization

**DOI:** 10.3389/fneur.2022.938333

**Published:** 2022-09-29

**Authors:** Yong-Feng Han, Peng Jiang, Zhong-Bin Tian, Xi-Heng Chen, Jian Liu, Zhong-Xue Wu, Bu-Lang Gao, Chun-Feng Ren

**Affiliations:** ^1^Department of Interventional Neuroradiology, Beijing Neurosurgical Institute and Beijing Tiantan Hospital, Capital Medical University, Beijing, China; ^2^Department of Neurosurgery, Shijiazhuang People's Hospital, Shijiazhuang, China; ^3^Zhengzhou University First Affiliated Hospital, Zhengzhou, China

**Keywords:** saccular aneurysms, second recurrence, embolization, risk factors, refractory

## Abstract

**Purpose:**

To explore the risk factors of recurrence after second endovascular embolization of recurrent aneurysms and the characteristics of recurrent refractory aneurysms to help clinical decision-making.

**Materials and methods:**

Forty-nine patients with recurrent aneurysms who underwent repeated embolization were retrospectively enrolled and divided into the recurrent and non-recurrent group. The risk factors of recurrence, complications and follow-up results of repeated embolization, and characteristics of recurrent refractory aneurysms were analyzed.

**Results:**

Among the 49 patients with the second embolization, 5 were lost to follow-up, 9 recurred, and 35 did not. Univariate analysis showed that aneurysm size (*P* = 0.022), aneurysm classification (*P* = 0.014), and Raymond-Roy grade after the second embolization (*P* = 0.001) were statistically different between the two groups. Multivariate analysis demonstrated the Raymond-Roy grade as an independent risk factor for the recurrence of aneurysms after the second embolization (*P* = 0.042). The complication rate after the second embolization was 4%. There were five recurrent refractory aneurysms with an average aneurysm size of 23.17 ± 10.45 mm, including three giant aneurysms and two large aneurysms. To achieve complete or near-complete embolization of the recurrent refractory aneurysms, multiple treatment approaches were needed with multiple stents or flow diverting devices.

**Conclusion:**

Aneurysm occlusion status after the second embolization is an independent risk factor for the recurrence of intracranial aneurysms. Compared with near-complete occlusion, complete occlusion can significantly reduce the risk of recurrence after second embolization. In order to achieve complete or near-complete occlusion, recurrent refractory aneurysms need multiple treatments with the use of multiple stents or flow diverting devices.

## Introduction

Ever since the short-, medium-, and long-term results of the International Subarachnoid Hemorrhage Trial (ISAT) were published (1–3), the proportion of endovascular embolization for intracranial aneurysms has been increasing (4–11). Endovascular embolization of cerebral aneurysms is no longer an alternative to surgical clipping, but one of the preferred treatment approaches (4, 5, 10–13). Compared with surgical clipping of aneurysms, interventional embolization has a lower mortality and disability rate (14–16), and after endovascular embolization, both the incidence of epilepsy and the ratio of cognitive impairment have decreased significantly (3, 17). Therefore, the 2012 American Stroke Association Guidelines on the diagnosis and treatment of aneurysmal subarachnoid hemorrhage pointed out that for patients with aneurysms suitable for both surgical clipping and interventional embolization, interventional embolization is recommended as the first choice (18). However, compared with surgical clipping, endovascular embolization has a higher recurrence rate, which is about 15–33% (19–26). Aneurysms need repeated treatment after recurrence; however, some aneurysms may recanalize again after the second embolization. At present, there is no unified viewpoint both on the factors affecting recurrence after second embolization and on the treatment method more suitable for retreatment. This retrospective study was performed to investigate the factors affecting recurrence after the second embolization and the follow-up outcomes for recurrent refractory aneurysms based on the data of aneurysmal patients in our department so as to provide some help for clinical decision-making.

## Materials and methods

This retrospective study was conducted between January 2012 and June 2017 with approval from the ethics committee of our hospital. All patients had given their signed informed consent to participate. The inclusion criteria were imaging-confirmed saccular cerebral aneurysms which were recurrent after endovascular embolization, the initial treatment and retreatment approaches being endovascular embolization and complete follow-up data in digital subtraction angiography (DSA). The exclusion criteria were dissection, fusiform, and blister-like aneurysms, pseudoaneurysms, aneurysms concurrent with a dural arteriovenous fistula, cerebral arteriovenous malformation, Moyamoya disease and malignant tumors, aneurysms which were treated with flow diverting devices at the first time, and aneurysms which were treated with parent artery occlusion at retreatment. The enrolled patients were divided into two groups: the recurrent group including aneurysms that were recanalized after the second endovascular embolization and the non-recurrent group with aneurysms that remained completely occluded after the second endovascular embolization.

The clinical data were collected, including age, sex, smoking history, alcohol abuse, hypertension, hyperlipidemia, and diabetes mellitus. The aneurysm data were also recorded, including the aneurysm size (giant aneurysms: >25 mm, large: 10–25 mm, medium: 5–10 mm, and small: <5 mm), wide or narrowed necks (wide: >4 mm or neck to dome ratio >1/2), lateral/bifurcation aneurysms, anterior or posterior circulation, and ruptured or un-ruptured aneurysms based on computed tomography (CT) findings of subarachnoid hemorrhage.

Aneurysm recurrence after the second endovascular embolization was defined as angiographically increased aneurysm volume at follow-up compared with angiography at the end of the second endovascular embolization. Non-recurrence was defined as no change in the aneurysm volume at follow-up angiography compared with that at the end of the second embolization. Aneurysm recurrence was confirmed by two experts in endovascular embolization. Mild recurrence indicated mild compression of coils with the display of the aneurysm neck, whereas major recurrence was caused by obvious loosening and compression of coils or herniation of coils with the display of the aneurysm dome. The Raymond-Roy classification system was used to describe aneurysm occlusion degree immediately after aneurysm embolization, with grade I indicating complete occlusion, II near complete occlusion with a residual neck, and III incomplete occlusion with a residual aneurysm sac. Recurrent refractory aneurysms refer to aneurysms that recur after the third endovascular embolization.

The re-embolization criteria were aneurysms with re-rupture, major recurrence or recurrence >20% of the original aneurysm volume, progressive enlargement of the remnant neck or growth of a small sac, and aneurysms that were suitable for both surgical clipping and endovascular embolization. For recurrent aneurysms after embolization, interventional embolization was preferred, especially for posterior circulation aneurysms. In order to prevent the recurrence after the second embolization, stent-assisted embolization should be performed as far as possible. If there was a high possibility of recurrence after stent-assisted embolization, multiple stents should be adopted to assist embolization, or flow diverting devices should be used. For recurrent aneurysms that were difficult to be treated by the second endovascular embolization or might not be embolized with complete or near-complete occlusion, surgical clipping should be considered.

Endovascular embolization was performed with the patient under general anesthesia. Systemic heparinization was applied during the procedure, with the first dose of heparin being 3,000 IU followed by 1,000 IU / h. The catheters and microcatheters were continuously infused with heparin sodium saline under pressure. After the right femoral artery was punctured using the Seldinger technique, a 6F or 8F femoral artery sheath was implanted. The guiding catheter was navigated to the level of the petrous bone segment of the internal carotid artery or the second cervical vertebra of the vertebral artery, and three-dimensional rotational angiography was performed with three-dimensional reconstruction for the measurement of recurrent aneurysm size and the proximal and distal diameters of the parent artery. Then, a microcatheter was navigated to the aneurysm cavity for coil embolization. Stent-assisted coiling was conducted for wide-necked aneurysms until no filling of contrast agent in the aneurysm. If the Pipeline Embolization Device (PED) was used to treat recurrent aneurysms, an 8F Envoy guiding catheter (Cordis, USA) and 6F Navien (Medtronic, USA) and Masksman (Medtronic, USA) microcatheters formed a triaxial system to support the release of PED.

For un-ruptured recurrent aneurysms requiring stent-assisted embolization, oral antiplatelet drugs (100 mg aspirin and 75 mg clopidogrel) were started at least 3 days before the operation. For recurrent aneurysms with acute rupture, aspirin (300 mg) and clopidogrel (300 mg) were administered orally or intranasally 2 h before the operation. For emergent treatment of ruptured aneurysms requiring stent-assisted embolization, tirofiban (8.0 μg / kg in a bolus within 3 min) was given after stent implantation, and tirofiban 0.1 μg / kg / min was continually pumped in after operation until the patient was able to take oral drugs. After the embolization, the patients were treated with dual antiplatelet medications (100 mg aspirin and 75 mg clopidogrel) for 6 weeks and then aspirin 100 mg for 6 months. For recurrent aneurysms that needed to be treated with PED, antiplatelet drugs (100 mg aspirin and 75 mg clopidogrel) were administered orally at least 5 days before the operation. Thromboelastogram was tested at the same time, and the drugs were adjusted according to the platelet activity inhibition rate. After the operation, the drugs were administered orally continuously for 6 months and then changed to aspirin 100 mg for life.

### Follow-up

Six months after the second embolization of intracranial aneurysms, whole-brain DSA was routinely performed for all patients, and then magnetic resonance angiography (MRA) or DSA was performed every 1 year according to the last angiographic follow-up results. The patients were followed up for at least 5 years. If follow-up DSA showed that the aneurysms had dense embolization with no obvious signs of recurrence, follow-up angiography would be performed with MRA. If follow-up DSA demonstrated recurrence signs in the embolized aneurysms, the follow-up would be performed with DSA.

All patients were evaluated with the modified Rankin scale (mRS) scores before discharge and 6 months after operation: 0, no symptoms or discomfort; (1) with symptoms but no neurological defect; (2) mild disability but being able to live on themselves; (3) moderate disability requiring other people's care; (4) moderate and severe disability, unable to take care of themselves; (5) severe disability and being bedridden; (6) death. The prognosis was good if the score was lower or stable between 0 and 2, but poor if the score was increased to 3–5.

### Statistical analysis

The statistical analysis was performed with the SPSS22.0 software (IBM, Chicago, IL, USA). Measurement data were expressed as mean ± standard deviation and tested with the independent sample *t*-test. Counting data were expressed as numbers and percentages and tested with the Chi-square test or Fisher's exact probability method. Univariate and multivariate logistic regression analyses were analyzed. *P* < 0.05 was considered to be statistically significant.

## Results

### Subjects

Among 867 patients with saccular cerebral aneurysms who were treated between January 2012 and June 2017, 123 patients experienced aneurysm recurrence (a recurrence rate of 14.2%), including 56 aneurysms with mild recurrence and 67 with major recurrence. Among 67 aneurysms with major recurrence, six patients were transferred to surgical clipping, and 12 refused retreatment because of a higher risk of treatment and poor prognosis. The remaining 49 patients with 49 recurrent aneurysms underwent the second endovascular embolization and were enrolled in this study, including 16 male and 33 female patients with an age range of 27–69 years (mean 47.71 ± 10.22). There were 18 patients with hypertension, 6 with hyperlipidemia, 5 with diabetes mellitus, 10 with alcohol abuse, and 7 with a smoking habit (Table 1).

**Table 1:** Baseline data of 49 patients with second endovascular embolization.

**Variables**	
Age (y)	47.71 ± 10.22
M/F	16/33
Hypertension/ Non-hypertension	18/31
Hyperlipidemia/ Non-hyperlipidemia	6/43
Diabetes mellitus/ Non-diabetes	5/44
Smoking history	7
Alcohol abuse	10

### Characteristics of recurrent aneurysms

The mean size of the 49 aneurysms was 13.07 ± 8.57 mm (range 3.37–38 mm), including 7 small, 16 medium, 21 large, and 5 giant aneurysms, with the large and giant aneurysms accounting for 53.1% (26/49). The mean size of the aneurysm neck was 4.76 ± 1.79 mm (range 1.3–9.37 mm), with 29 wide-necked and 20 narrow-necked aneurysms. Twenty-four aneurysms were ruptured, 29 lateral and 44 in the anterior circulation. There were 18 aneurysms in the ophthalmic artery segment: 14 in the posterior communicating artery segment, 5 at the anterior communicating artery, 4 in the cavernous segment, 2 at the basilar artery, 1 at the middle cerebral artery, and 5 in other locations (Table 2).

**Table 2:** Baseline data of 49 aneurysms.

**Variables**	
Aneurysm size (mm)	13.07 ± 8.57
Small	7
Medium	16
Large	21
Giant	5
Aneurysm neck (mm)	4.76 ± 1.79
Wide/narrow neck	29/20
Ruptured/ Non-ruptured	24/25
**Aneurysm location**	
Anterior/posterior circulation	44/5
Anterior communicating artery	5
Posterior communicating artery	14
Middle cerebral artery	1
Ophthalmic artery	18
Cavernous segment	4
Basilar artery tip	2
Other locations	5
Lateral/bifurcation	29/20

### Risk factors for aneurysm recurrence

**Figure 1:**
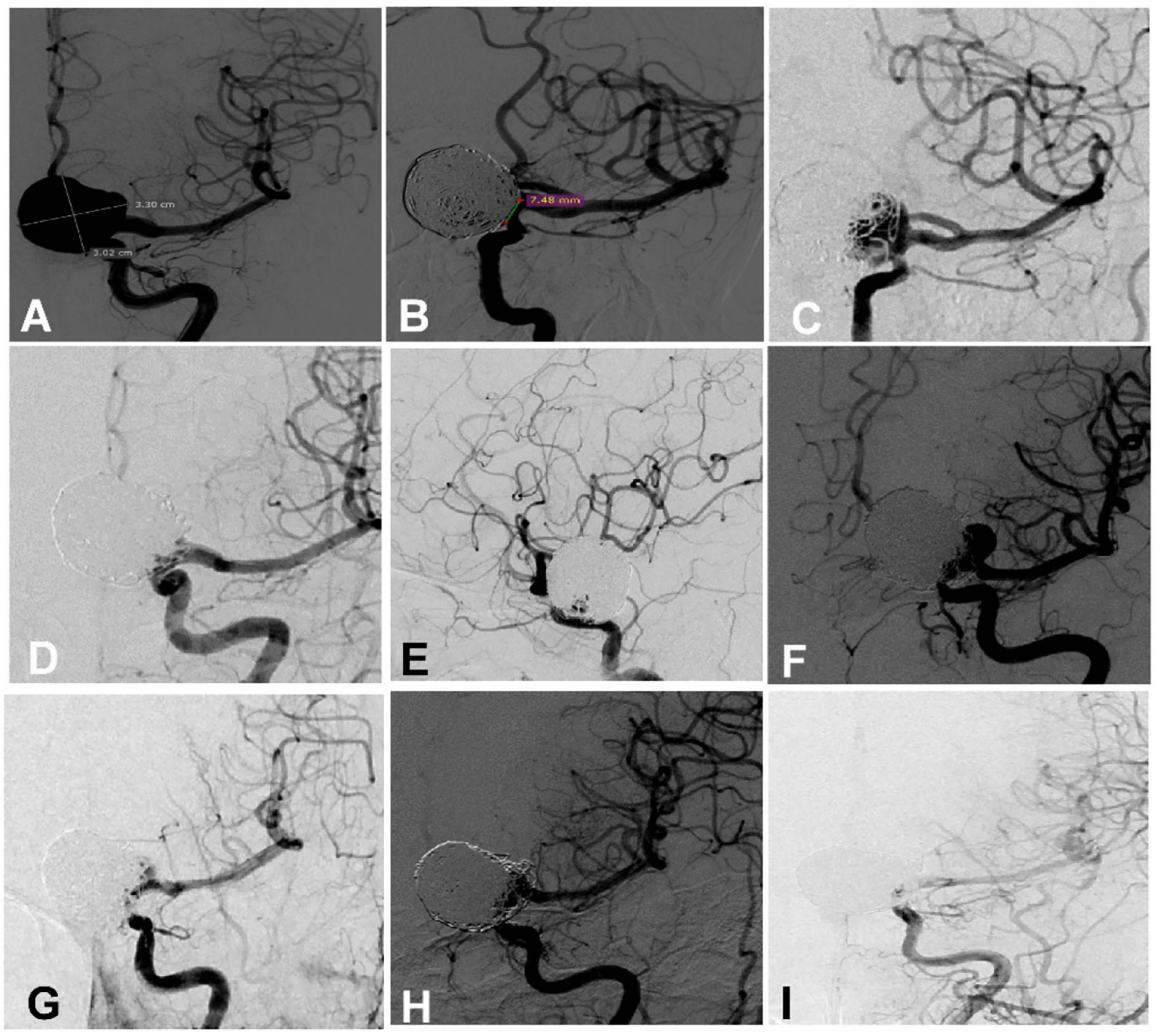
A woman in her 50 s had a giant aneurysm on the left ophthalmic artery segment which was treated multiple times. **(A)** Cerebral angiography revealed a giant aneurysm measuring 33.0 × 30.2 mm with a 7.48 mm neck. **(B)** The aneurysm was embolized with an Enterprise stent to assist coiling, resulting in near complete occlusion at the end of embolization. **(C)** Follow-up angiography 9 months later revealed recurrence of the aneurysm. **(D,E)** The recurrent aneurysm was treated with the deployment of an Enterprise stent to assist coiling, and near complete occlusion was achieved at the end of embolization. **(F)** Eight months later, the aneurysm was recurrent again. **(G)** At the third embolization, an Enterprise stent was deployed to assist coiling, resulting in near complete occlusion. **(H)** Four-month angiographic follow-up revealed that the aneurysm occlusion was stable. **(I)** Follow-up 1 year later revealed that the aneurysm was completely occluded.

**Figure 2:**
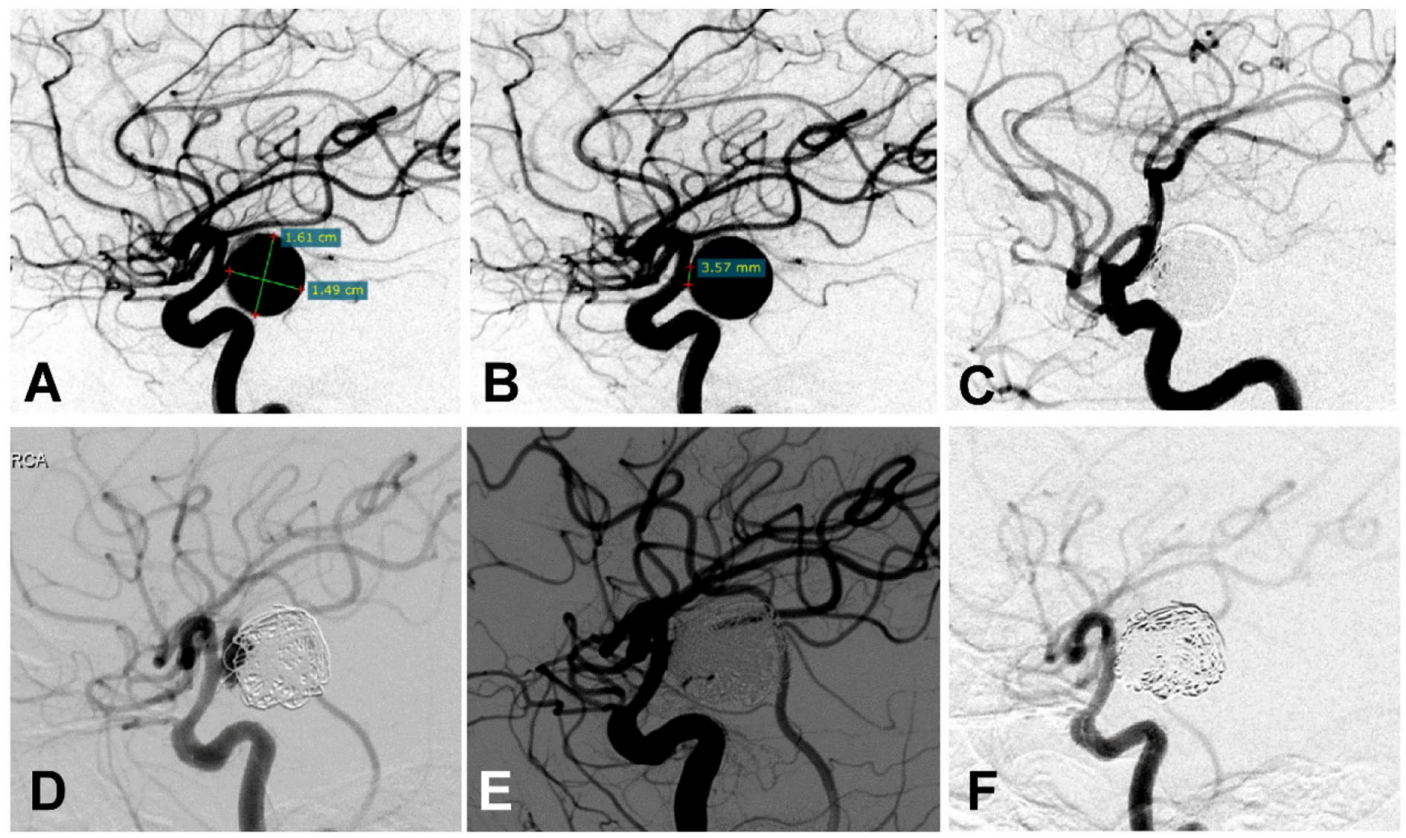
A woman in her 50 s had a posterior communicating artery (Pcom) aneurysm on the right side treated multiple times. **(A,B)** Cerebral angiography before treatment showed the Pcom aneurysm measuring 16.1 ×14.9 mm with a 3.57 mm neck. **(C)** The aneurysm was treated with an Enterprise stent to assist coiling, and the aneurysm was completely occluded immediately after the embolization. **(D)** Six months later, cerebral angiography revealed recurrence of the aneurysm. **(E)** The recurrent aneurysm was re-treated with a Low-Profile Visualized Intraluminal Support (LVIS) stent plus coiling, resulting in complete occlusion of the aneurysm immediately at the end of embolization. **(F)** Six months later, cerebral angiography showed dense embolization without recurrence.

After the second endovascular embolization of recurrent aneurysms, 5 patients were lost to follow-up, and the remaining 44 patients had angiographic follow-up and were divided into the recurrent (*n* = 9, Figure 1) and non-recurrent (*n* = 35, Figure 2) groups.

The clinical data (age, sex, hypertension, hyperlipidemia, and diabetes mellitus), aneurysm data (aneurysm size, neck size, type, location, and rupture status), re-embolization approaches, follow-up time, and aneurysm occlusion status were analyzed. Univariate analysis demonstrated a significant (*P* < 0.05) difference between the two groups in aneurysm size (*P* = 0.022), aneurysm classification based on aneurysm size (*P* = 0.014), and aneurysm occlusion degree (Raymond-Roy classification system, *P* = 0.001) after the second embolization (Table 3). No significant (*P* > 0.05) difference was detected between the two groups in age, sex, hypertension, hyperlipidemia, diabetes mellitus, aneurysm neck size, aneurysm type and location, rupture status, mode of second embolization, aneurysm occlusion degree after the first embolization, and follow-up time.

**Table 3:** Univariate analysis of risk factors affecting recurrence after second embolization.

**Risk factors**	**Non-recurrent (*n* = 35)**	**Recurrent (*n* = 9)**	**(χ^2^/t)**	***P***
Age (y)	46.71 ± 9.75	46.56 ± 10.61	−0.043	0.966
M	12	3	0.003	1.00
F	25	6		
Hypertension	14	3	0.134	1.00
Hyperlipidemia	5	1	0.061	1.00
Diabetes mellitus	4	1	0.001	1.00
Aneurysm size (mm)	10.28 ± 5.88	16.58 ± 10.80	2.380	0.022
Small	7	0	9.704	0.014
Medium-sized	13	3		
Large	15	3		
Giant	0	3		
Aneurysm neck (mm)	4.38 ± 1.77	5.43 ± 1.84	1.575	0.123
Wide neck	16	8		
Narrow neck	19	1		
Aneurysm type			0.670	0.477
Lateral	18	6		
Bifurcation	17	3		
Ruptured	20	4	0.466	0.71
Non-ruptured	15	5		
Anterior circulation	31	8	0.001	1.00
Posterior circulation	4	1		
Second embolization mode			4.457	0.099
Coiling alone	11	6		
Stent-assisted coiling	21	2		
PED	3	1		
Raymond-Roy grade after 2^nd^ embolization			12.394	0.001
I	26	1		
II	7	7		
III	2	1		
Follow-up duration (m)	12.2 ± 8.97	14.38 ± 19.01	0.296	0.769
Raymond-Roy grade after 1^st^ embolization			1.1	0.655
I	13	2		
II	20	7		
III	2	0		

After the risk factors with *P* < 0.1 in the univariate analysis were included in the multivariate analysis, including aneurysm size, embolization method, and Raymond-Roy grade after the second embolization, the Raymond-Roy grade was an independent risk factor for the recurrence of intracranial aneurysms after the second embolization (*P* = 0.042) (Table 4). Compared with near-complete occlusion, complete embolization significantly reduced the risk of recurrence after second embolization.

**Table 4:** Multivariate analysis of risk factors affecting recurrence after second embolization.

**Risk factors**	**β**	**Standard error**	**OR**	**95% CI**	***P***
Aneurysm size (mm)	−0.126	0.227	0.881	0.565–1.374	0.577
**Second embolization mode**					
Coiling alone					
Stent-assisted coiling	−1.617	1.069	0.198	0.024–1.614	0.13
PED	−1.518	1.794	0.219	0.007–7.375	0.397
**Raymond-Roy grade after second embolization**					
I					
II	2.614	1.287	13.656	1.096–170.206	0.042

OR, odds ratio; CI, confidence interval; PED, Pipeline Embolization device.

### Procedural complications

Two patients (4%) had a thrombotic event during the second embolization procedure of recurrent aneurysms, without causing any symptoms. In one case of internal carotid artery ophthalmic artery aneurysm treated with stent-assisted embolization, stent thrombosis occurred, and 10 ml of tirofiban (0.1 mg / ml) was given intravenously rapidly and later was pumped in continuously for 24 h (4 ml / h, 0.1 mg / ml). After the blood vessel was recanalized, neither new cerebral infarction nor neurological deficit was found in the postoperative examination. In the other case harboring an A1 aneurysm at the anterior cerebral artery which was embolized with coils alone, the coil herniated into the parent artery, resulting in ipsilateral A1 occlusion but no symptoms because of collateral circulation through the opened anterior communicating artery. There were no bleeding events during and 1 month after the second embolization.

### Follow-up outcomes and retreatment

Among the 49 patients with recurrent aneurysms treated with the second embolization, five cases were lost to follow-up, and the remaining 44 cases were followed up for an average of 12.45 ± 11.14 months, with recurrence in nine cases, including three cases of mild recurrence and six cases of major recurrence. Five patients with major recurrence received the third endovascular embolization (Figure 1). Among 44 patients who were followed up for 6 months after the operation, no patients had increased mRS scores.

Among 49 patients with recurrent aneurysms who received repeated embolization, five aneurysms in five patients received embolization≥3 and were defined as recurrent refractory aneurysms (two ruptured and three un-ruptured), with the aneurysm size ranging from 14.8 mm to 38 mm (mean 23.17 ± 10.45), including three giant aneurysms and two large aneurysms. There were four women and one man with an age range of 36–60 years (mean 49.4 ± 9.42). The location of the recurrent refractory aneurysms was the ophthalmic artery segment in three cases, a posterior communicating artery in one, and the A1-A2 junction in one. Four patients received embolization three times, and one received embolization four times. Eleven Enterprise stents and two PEDs were used in treating these five recurrent refractory aneurysms (Tables 5, 6).

**Table 5:** Baseline data of recurrent refractory aneurysms.

**Cases**	**Age (y)**	**Sex**	**Rupture**	**Maximal diameter (mm)**	**Location**	**Embolization frequency**	**Stent**
1	44	F	No	26.3	Ophthalmic segment	4	5 En
2	53	F	No	38	Ophthalmic segment	3	3 En
3	54	F	Yes	11.64	Ophthalmic segment	3	2 En
4	60	F	No	25.1	Pcom	3	1En&1 PED
5	36	M	Yes	14.8	A1-A2 junction	3	1 PED

En, Enterprise stent; Pcom, posterior communicating artery; PED, Pipeline Embolization device.

**Table 6:** Embolization method and occlusion degree immediately after embolization of recurrent refractory aneurysms.

**Cases**	**1^st^ embolization**	**2^nd^ embolization**	**3^rd^ embolization**	**4^th^ embolization**
1	En+coils, near complete	En+coils, near complete	Coils alone, near complete	3En+coils, complete
2	En+coils, near complete	En+coils, near complete	En+coils, near complete	
3	En+coils, near complete	Coils alone, near complete	En+coils, complete	
4	En+coils, near complete	Coils alone, near complete	PED, incomplete	
5	Coils alone, near complete	Coils alone, near complete	PED, incomplete	

En, Enterprise stent; PED, Pipeline Embolization device.

## Discussion

### Major findings

With continuous progress in endovascular treatment technology and concept, as well as rapid development in new materials and equipment, endovascular treatment technology has been widely used in the treatment of intracranial aneurysms. Compared with surgical clipping, interventional embolization of aneurysms has the advantages of less trauma, faster recovery, and lower surgical risk, and has been accepted by the majority of patients as one of the first choices for the treatment of intracranial aneurysms (25, 27–31). However, the recurrence rate was significantly higher than that of surgical clipping (2, 19–21), with the recurrent ruptured hemorrhage of embolized aneurysms characterized by high mortality and disability, requiring repeated endovascular embolization. Nonetheless, some aneurysms continued to recur after the second embolization, and recurrent aneurysms are more difficult to treat and have a serious impact on patients and their families. Therefore, it is of great significance to explore the factors affecting the recurrence of intracranial aneurysms after endovascular embolization. In our study, univariate analysis revealed aneurysm size (*P* = 0.022) and aneurysm occlusion degree (*P* = 0.001) as the risk factors affecting recurrence after the second embolization, whereas multivariate analysis demonstrated the Raymond-Roy aneurysm occlusion grade as an independent risk factor affecting the recurrence of intracranial aneurysms after the second embolization (*P* = 0.042). Complete occlusion significantly reduces the risk of recurrence after second embolization compared with near-complete occlusion. Multiple stents or PEDs are needed to completely occlude the recurrent refractory aneurysms.

### Retreatment principles and method choice

At present, there is no consensus on the principle of retreatment for recurrent aneurysms after embolization. Raymond et al. (20) suggested that aneurysms with a mild recurrence should be observed continuously, while aneurysms with major recurrence or re-rupture should be treated as soon as possible. The CARAT study (32) has revealed that the residual grade of ruptured aneurysms after embolization was significantly correlated with the rebleeding rate of Raymond grades II and III, and the average rebleeding rate was 2.9 and 5.9%, respectively, at 4-year follow-up. Therefore, it is suggested that the aneurysms of Raymond-Roy grade III after embolization should be re-treated as soon as possible (32). Plowman et al. (33) reported the follow-up results of 16 years after embolization of ruptured aneurysms, and they found that recurrent aneurysms and rebleeding mostly occurred in unstable aneurysms of Raymond-Roy grades II and III, suggesting that such aneurysms should be re-treated. We followed the principles of retreatment for recurrent intracranial aneurysms after embolization suggested by Dorfer et al. (34): (1) the residual aneurysm was larger than 20% of the initial aneurysm volume; (2) the aneurysmal remnant neck was unstable and enlarged progressively; (3) a new sac had grown on the aneurysm; and (4) the aneurysm was enlarged (not caused by coil compression).

At present, there is no unified view on the method of retreatment. Dorfer et al. (34) believed that the principle of individualization should be followed in the retreatment: the age and physical condition of each patient, the size and location of each aneurysm, the size of the residual aneurysm, the presence or absence of coils in the neck of the aneurysm, the mechanism of aneurysm recurrence, and the history of subarachnoid hemorrhage should all be taken into consideration.

In our study, we usually give priority to interventional therapy for recurrent aneurysms after embolization, especially for posterior circulation aneurysms. For recurrent refractory aneurysms, we even adopted multiple stents to assist embolization or used the flow diverting device for treatment. For recurrent aneurysms that are very difficult to be re-treated by endovascular intervention or may not be completely or nearly completely embolized, surgical clipping is recommended. Large or giant aneurysms with a wide neck or a perforator are prone to recurrence after repeated endovascular embolization. For large or giant wide-necked aneurysms, stent-assisted coiling may not be able to obtain an endurable complete occlusion status, and flow diverters with adjunctive coiling are preferred. For large or giant aneurysms with a perforator, aneurysm recurrence is easy because dense packing cannot be achieved for fear of occluding the perforator, and in these cases, surgical clipping is recommended. Nonetheless, the morphology and position of the aneurysm should be considered in repeated treatment. For recurrent aneurysms, especially those in the posterior circulation, we tended to use endovascular embolization in the repeated treatment. This is because the risk and difficulty of surgical clipping are greatly increased for recurrent aneurysms which had been treated with coil embolization, especially for those at the posterior circulation. For one thing, coils within the aneurysm sac may cause the surgical clip to shift, resulting in stenosis or occlusion of the parent artery. Clip shift may increase the difficulty to free the aneurysm, which increases the difficulty of surgical clipping. For another, due to the space-occupying effect of the coils inside the aneurysm sac, the operation space for clipping is limited. Even in some cases, the coils are closely related to the aneurysm neck so that the aneurysm neck cannot be directly clamped. At this time, it is necessary to remove part or all of the coils, which are closely adhered to the aneurysm wall. Particularly, when the aneurysm neck is involved, forcibly removing the coils may lead to tearing of the aneurysm neck, resulting in serious consequences.

### Risk factors affecting recurrence after the second embolization

Because the number of recurrent cases after embolization is usually small, there is little literature about the risk factors affecting the recurrence of intracranial aneurysms after the second embolization. Some authors have reported a few cases of major recurrence of aneurysms after the second embolization but without analyzing the risk factors affecting the recurrence (35, 36). Henkes et al. (37) reported a large series of patients with 145 aneurysms receiving three or more times of embolization treatments, but they did not analyze the risk factors affecting the recurrence after repeated treatment. Lee et al. (38) reported that a total of 133 aneurysms in 129 patients received the second embolization because of recurrence, with 47 aneurysms being recurred two times at a 6-month follow-up. Multivariate analysis showed that large aneurysms (>7 mm), incomplete embolization of aneurysms after the second treatment, and posterior circulation aneurysms were the influencing factors of the second recurrence. In 2012, Cho et al. (39) reported their 10-year research results, and they found that large aneurysms, stent application, and incomplete embolization were risk factors for the second recurrence of aneurysms. In our study, it was found that aneurysm size (*P* = 0.022) and aneurysm occlusion degree (*P* = 0.001) were the risk factors affecting recurrence after the second embolization. Generally, large aneurysms have relatively wide necks. After recurrence, the neck is relatively shallow and difficult to embolize, and incomplete occlusion may result after embolization, which is a possible reason for repeated recurrence (38). Moreover, aneurysms with wider necks usually have larger blood flow and complex stress gradient, which will lead to coil compression and repeated recurrence.

### Retreatment rate and complications of second embolization

According to the literature, the recurrence rate of intracranial aneurysms after embolization is 6.1–33.6% (15, 19, 20). However, the retreatment rate of recurrent aneurysms is low, about 4.7–17.4% (34). In a meta-analysis conducted by Ferns et al. with a total of 46 studies involving 8,161 aneurysms (19), 21% of the aneurysms recurred with 10% of the aneurysms being treated again at follow-up. In the CARAT study, the retreatment rates of aneurysms after interventional embolization in the first year and the second year were 13.3 and 4.5%, respectively (40). According to the International Subarachnoid Hemorrhage experiment (ISAT), 191 out of 1,096 aneurysms received retreatment during an average follow-up of 5.7 months, with a retreatment rate of 17.4% (2).

It is safe to treat recurrent aneurysms with interventional embolization, and the complication rate is very low. Kang et al. (35) and Slob et al. (36) reported that the mortality and disability rates of recurrent aneurysms after repeated interventional embolization were zero. Renowden et al. (41) reported that the incidence of mild neurological dysfunction was 3% in 100 recurrent aneurysms with repeated treatment. Henkes et al. (37) reported 350 recurrent aneurysms being treated again, and among 495 times of retreatment, 11 cases had complications, resulting in a complication rate of 2.2%. Ringer et al. (42) reported the results from eight research centers in the United States and Puerto Rico, and 311 patients with recurrent aneurysms received 352 times of interventional embolization, with the probability of death and permanent disability in each treatment being 1.13%.

### Treatment of refractory recurrent aneurysms

Refractory aneurysms indicated those of (1) giant aneurysms (> 2.5 cm), accompanied by peripheral nerve and vascular compression symptoms; (2) fusiform and serpentine aneurysms, with wide necks or undefined necks; and (3) atherosclerotic plaques in the aneurysm (43). Complex and refractory aneurysms also included the following types: giant aneurysms (often with thrombus or sclerotic plaques), serpentine or fusiform aneurysms by morphology, pseudoaneurysms, dissecting aneurysms, and blood-bubble-like aneurysms by aneurysm wall structure (44). In our study, refractory recurrent aneurysms were defined as aneurysms with more than three times endovascular treatment. Five patients with refractory recurrent aneurysms were included in our study, with the following characteristics: (1) giant aneurysms in three cases and large aneurysms in two, with the average size of aneurysms of 23.17 ± 10.45 mm (range 14.8–38 mm); (2) multiple treatment approaches were needed with multiple stents or flow diverting devices in order to cure the aneurysms.

Chalouhi et al. (45) reported six patients with complex refractory aneurysms which were treated with multiple stents, with three Neuroform stents and one Enterprise stent being applied to one aneurysm at a time. In the 9-month follow-up results, six cases of aneurysms achieved complete or near-complete embolization. They consequently believed that overlapping Neuroform and Enterprise stents may induce complete thrombosis of intracranial aneurysms and facilitate parent artery remodeling. After comparing the long-term follow-up imaging results of the single stent and overlapping stents in the treatment of intracranial aneurysms, with an average follow-up of 16.21 months, Yoon et al. (46) found that the average occlusion rate of aneurysms treated with a single stent was only 64.41%, while that with overlapping stents was 98.57%. The average occlusion rate of intracranial aneurysms treated with overlapping stents was significantly higher than that of single stent treatment (*P* = 0.005).

Hemodynamic studies also showed that the flow velocity into intracranial aneurysms and the shear stress of aneurysm wall both decreased with the number of stents deployed within the parent artery (47, 48), indicating that multiple stents have more advantages than a single stent in the treatment of complex aneurysms. Compared with a single stent, the mesh of multiple stents is further reduced, which makes the hemodynamic changes more obvious, consequently promoting the formation of thrombus in aneurysms and repairing the intima of the vascular wall better for complete occlusion of the aneurysm. Thus, blood flow diverting devices came into being.

The blood flow diverting device has a higher metal coverage rate, can better divert the blood flow away, and more easily promote the vascular endothelium to cover the damaged area of the parent artery, so as to achieve the purpose of curing the aneurysm. It transfers the idea of treating aneurysms from the filling of aneurysms to the reconstruction of the parent artery, which makes many complex aneurysms, such as serpentine and fusiform aneurysms, cured. Some good results have been achieved in using the PED for treating recurrent aneurysms with a complete occlusion rate between 76.7 and 86.7% at follow-up (49–51). The flow diverting device may thus be an alternative treatment for recurrent refractory aneurysms because it can not only promote the formation of thrombosis within aneurysms by blocking blood flow, but can also remodel the parent artery through its own network structure to promote vascular endothelium regeneration, thus completely excluding the aneurysm from the parent artery.

### Limitations

Some limitations existed in this study. First, the compliance of the patients was poor, and regular angiographic follow-up could not be completed on time. Second, the sample size of this study was relatively small. Third, this study was a single-center, retrospective study. All these issues may affect the publication bias, and the results of this study need to be further verified by multiple centers with prospective randomized controlled studies.

## Conclusion

In conclusion, the Raymond-Roy grade of aneurysm occlusion status after the second embolization is an independent risk factor for the recurrence of intracranial aneurysms. Compared with near-complete occlusion, complete occlusion can significantly reduce the risk of recurrence of aneurysms after second embolization. In order to achieve complete or near-complete occlusion, recurrent refractory aneurysms need multiple treatments with the use of multiple stents or flow diverting devices.

## Data availability statement

The original contributions presented in the study are included in the article/supplementary material, further inquiries can be directed to the corresponding author.

## Ethics statement

The studies involving human participants were reviewed and approved by Ethics Committee of Beijing Tiantan Hospital. The patients/participants provided their written informed consent to participate in this study.

## Author contributions

Y-FH and PJ: study design. Y-FH, Z-BT, X-HC, JL, and Z-XW: data collection. Y-FH and B-LG: data analysis. Y-FH: writing of the original work and supervision. B-LG: review and revision. All authors contributed to the article and approved the submitted version.

## Conflict of interest

The authors declare that the research was conducted in the absence of any commercial or financial relationships that could be construed as a potential conflict of interest.

## Publisher's note

All claims expressed in this article are solely those of the authors and do not necessarily represent those of their affiliated organizations, or those of the publisher, the editors and the reviewers. Any product that may be evaluated in this article, or claim that may be made by its manufacturer, is not guaranteed or endorsed by the publisher.
